# Impact of adjusted kidney volume measured in the bench surgery on one-year renal function in kidney transplantation

**DOI:** 10.1371/journal.pone.0224364

**Published:** 2019-11-04

**Authors:** Flávio Vasconcelos Ordones, Pedro Ivo Rocchetti Pajolli, Rodrigo Guerra da Silva, Hamilto Akihissa Yamamoto, Fernando Fereira Gomes Filho, Paulo Roberto Kawano, João Luiz Amaro, Luis Gustavo Modelli de Andrade

**Affiliations:** 1 Department of Urology, Botucatu Medical School, São Paulo State University (Universidade Estadual Paulista–UNESP), Botucatu, São Paulo, Brazil; 2 Urology Department, Royal Adelaide Hospital, Adelaide, South Australia, Australia; 3 BP Hospital, São Paulo, Brazil; 4 Hospital 9 de Julho, SP, Brazil; 5 Sirio Libanes Hospital, SP, Brazil; 6 Department of Internal Medicine, Nephrology, Botucatu Medical School, São Paulo State University (Universidade Estadual Paulista–UNESP), Botucatu, São Paulo, Brazil; The George Institute for Global Health, UNSW, AUSTRALIA

## Abstract

**Background:**

Kidney transplantation is the treatment of choice in patient with end stage chronic kidney disease, offering the best long term survival and greater Quality of Life in this group of patients. Graft volume was correlated with improved renal function in living donor transplantations. The primary aim of this study was to correlate renal volume adjusted to body surface area with renal function one year (estimated glomerular filtration rate; eGFR) after kidney transplantation.

**Methods:**

This single-center, prospective cohort study included 256 patients who underwent kidney transplantation from January 2011 through December 2015 at Hospital das Clínicas de Botucatu–UNESP. We evaluated three kidney measurements during the bench surgery; the final graft volume was calculated using the ellipsoid formula and adjusted to body surface area.

**Results:**

In the living donors there was positive correlation between adjusted graft volume and eGFR (r = 0.311, p = 0.008). Multivariate analysis revealed that low rejection rate and increased adjusted graft volume were independent factors correlated with eGFR. In deceased donors, there was no correlation between adjusted kidney volume and eGFR (r = 0.08, p = 0.279) in univariate analysis, but a multivariate analysis indicated that lower kidney donor profile index (KDPI), absence of rejection and high adjusted kidney volume were independent factors for better eGFR.

**Conclusion:**

Adjusted kidney volume was positively correlated with a satisfactory eGFR at one year after living donor and deceased donor transplantations.

## Introduction

End-stage renal disease is an increasingly prevalent public health problem [1,2]. Currently, kidney transplantation is the best therapeutic indication for patients with end-stage renal disease; transplantation is associated with better quality of life and survival compared with dialysis [3].

Although improvements in immunosuppressive regimes have resulted in significant improvements in early renal function [4], long-term graft survival remains suboptimal. Several factors potentially affect kidney survival, including donor organ quality and kidney volume [5,6]. Larger kidneys have higher glomerular filtration rates, which result in better renal function. Previous studies have shown that a decrease in kidney mass may lead to hyperfiltration, causing albuminuria and glomerulosclerosis. These results suggest that the number of nephrons or “nephron dose” of the graft may be a contributing factor to graft function [7–9].

Graft volume and/or mass are correlated with improved renal function in living donor transplantations [10–17]. On the other hand, results from deceased donor transplantations are controversial [18–21]. In most studies, kidney volume measurements were obtained via tomography [10–14], magnetic resonance imaging or ultrasound [18]. Although kidney volume has already been shown to be relevant to have a better transplant outcome, this measure has not been applied because estimating kidney volume requires complex formulas. As a result, the adoption of these techniques in daily clinical practice has remained unattractive [22].

Kidney volume can be estimated using three kidney measurements: width, length and thickness [23]. These dimensions can easily be measured by a surgeon at organ procurement or immediately prior to transplantation.

The primary aim of this study was to correlate renal volume adjusted to body surface area with renal function one year after transplantation.

## Materials and methods

This single-center, prospective cohort study was conducted at the School of Medicine of São Paulo State University (UNESP). The study was approved by the local research ethics committee (Comitê de Ética em Pesquisa–CEP FMB UNESP–request number 986.459). Written informed consent was obtained from all patients. All patients who underwent living or deceased donor renal transplantation between January 2011 and December 2015 were prospectively evaluated. Patients with less than one year of follow-up, those without kidney measurements, and those younger than 18 years of age were excluded. Donor allocation was based on human leukocyte antigen (HLA) compatibility. For deceased donors, allocation was determined according to blood type and HLA compatibility. For living donors, HLA compatibility was considered. This situation is in compliance with Brazilian legislation, which allows for donations between relatives up to the fourth degree. Study protocols for living donors are based on two measurements of glomerular filtration (i.e., creatinine clearance and the estimated glomerular filtration rate). We analyzed images using contrast angiotomography to evaluate kidney abnormalities. We excluded donors with abnormalities in kidney function (estimated glomerular filtration rate; eGFR < 90ml/mi), albuminuria (>30mg/g), hypertension, diabetes, a body mass index (BMI) exceeding 32 kg/m^2^, microscopic hematuria, parenchymal or urological abnormalities or nephrolithiasis.

### Kidney volume estimation

During the bench surgery, kidneys from living or deceased donors were perfused and prepared for transplantation. Excess fat was removed to enable adequate inspection of the organ and to accurately define the renal outline. Craniocaudal (length), laterolateral (width) and anteroposterior (thickness) measurements, expressed in centimeters (cm), were made using a graduated ruler (S1 Fig). The fat was carefully excluded from these measurements, as described by Kang et al. [24].

The measurements were always performed by a permanent member of the surgical team; these individuals have performed a similar number of transplantations. The final graft volume was calculated using the three measurements taken and the ellipsoid formula. The final result was expressed in cubic centimeters (cm^3^) according to the formula:

$$Ellipsoid\;volume\;({cm}^{3})=\frac{4}{3}\pi \times length\;(cm)x\;thickness(cm)$$

The kidney volume estimate was then corrected using body surface area (adjusted to 1.73 m^2^), as proposed by Poggio et al. [10]:

$$Adjusted\;Kidney\;Volume\;\left(\frac{{cm}^{3}}{1.73\;m^{2}}\right)=\frac{Elipsoid\;Kidney\;Volume\times 1.73}{Recipient\;Body\;Surface\;(m^{2})}$$

Body surface area was calculated using the DuBois formula, where X is weight in kilograms and Y is height in centimeters:

$$Body\;Surface\;Area\;(m^{2})=0.007181\times x^{0.425}\times y^{0.725}$$

### Immunosuppression

The combination of tacrolimus with mycophenolate and prednisone was used in living and deceased donor transplantations to obtain tacrolimus serum levels of 8–10 ng/ml in the first month and 4–8 ng/ml afterwards. Immunosuppression induction was performed using basiliximab or thymoglobulin at a dose of 3 mg/kg. This protocol is the immunosuppression standard of care with a combination of tacrolimus associated with mycophenolate and induction therapy [25]. The induction therapy was selected according to hospital availability; there was no additional risk of rejection based on using basiliximab or low-dose thymoglobulin. A dose of 6 mg/kg thymoglobulin was used in recipients with panel reactivity higher than 50%. For living donors, induction therapy was not used until 2014; an exception was made for patients with panel reactive antibody greater than 50. We changed the protocol of living donors by adding induction therapy for all patients in 2014 because of the higher incidence of T-cell-mediated rejection [26]. We did not perform transplants when the mean fluorescence intensity (MFI) of anti-donor antibodies exceeded 1500 or in cases of a positive cytotoxic crossmatch.

### Clinical variables

Demographic data, including age, weight, height, BMI, body surface area, sex and ethnicity were collected at the time of transplantation. Other analyzed variables included baseline disease status, time prior to transplantation, induction therapy and immunosuppressive regimen. The antibody reactivity panel (Panel), the number of HLA mismatches and re-transplantation cases were analyzed to assess immunological risk.

### Donor data

Donor age and sex were analyzed for living donor transplantations. For deceased donor transplantations, we also evaluated the presence of arterial hypertension and diabetes, the cause of donor death, the final creatinine and the total cold ischemia time.

### One-year follow-up

The number of biopsy-proven acute rejection and cytomegalovirus infection episodes were evaluated over the course of one year.

### Estimated glomerular filtration rate

The eGFR was calculated from creatinine levels assessed one year after transplantation using formula 4 of the Modification of Diet in Renal Disease (MDRD), according to Levey [27]:

$$eGFR=175\times {Creatinine}^{-1.154}\times {Age}^{-0.203}\times 1.212\;(if\;afrodes)\times 0.742\;(if\;woman)$$

### Statistical analysis

The Kolmogorov-Smirnov test was used to define the parametric and non-parametric distribution pattern of continuous variables. Parametric continuous variables were expressed as means and standard deviations, and non-parametric variables were expressed as medians with 25th and 75th percentiles. The Pearson product-moment correlation coefficient was used for living donor transplantations to assess correlations between the eGFR at the one-year follow-up and renal volume adjusted for body surface area. Spearman’s rank correlation was used for this analysis for deceased donor transplantations. The analyses of living and deceased donors were performed separately. For both living and deceased donors, a linear regression model was constructed for possible confounders associated with eGFR. The Stepwise model was used for multivariate linear regression analysis with a variable entry criterion of p = 0.15 and a variable removal criterion of p = 0.20. The following predictor variables were considered: age, sex, BMI, baseline disease, length of dialysis time, mismatches, panel reactive antibody count, immunosuppression, induction therapy, presence of rejection, cytomegalovirus and adjusted kidney volume. For deceased donors, the cold ischemia time and donor characteristics were also considered, including a kidney donor profile index (KDPI). Because of the higher degree of multicollinearity between donor variables of age, cause of death, hypertension, creatinine, ethnicity, and gender we only included the KDPI in the regression models. eGFR prediction equations were constructed based on multivariate linear regression analysis. A receiver operating characteristic (ROC) curve was constructed using eGFR at the one-year follow-up; eGFR exceeding 60 ml/min was used as a reference and adjusted kidney volume was used as the test variable. The best cut-off value for kidney volume was assessed based on the largest sum of sensitivity and specificity.

## Results

### Living donor transplantations

A total of 71 of the 256 transplantations included in the analysis were living donor transplantations. The mean age was 37 ± 11 years, and the recipients were predominantly white males. The most prevalent baseline disease was glomerulopathy, followed by indeterminate causes. Patients had low immunological risk, with a median panel reactivity of zero. Induction therapy was performed using basiliximab or thymoglobulin (3 mg/kg) in most cases. The predominant immunosuppressive regimen was a combination of tacrolimus, mycophenolate and prednisone. The one-year rejection rate was 26.8%, and the rate of cytomegalovirus infection was 18.6%. The mean kidney volume adjusted for body surface was 151.69 ± 41.66 (cm^3^/1.73 m^2^). The mean GFR at the one-year follow-up was 64.8 ± 23.9 ml/min (Table 1).

**Table 1:** Patient characteristics at baseline and after the one-year follow up–the living donor kidney transplantation group (n = 71) and the deceased donor group (n = 185).

		Living (n = 71)	Deceased (n = 185)
**Age (year)**		37±11	50±13
**Male gender n (%)**		43 (60.6%)	117 (63.2%)
**Body mass index (kg/m**^**2**^**)**		24.8 ± 4.8	25.7 ± 5.0
**Body surface (kg/m**^**2**^**)**		1.77 ± 0.19	1.77 ± 0.20
**Race**	White n (%)	44 (62.0%)	123 (66.5%)
	Mixed-race n (%)	20 (28.2%)	44 (23.8%)
	African American n (%)	6 (8.5%)	18 (9.7%)
	Asian n (%)	1 (1.4%)	0 (0%)
**Baseline Disease**	Hypertensionn (%)	7 (9.9%)	51 (27.8%)
	Diabetes n (%)	4 (5.6%)	45 (24.3%)
	Glomerulonephritis (%)	24 (33.8%)	24 (13.0%)
	Unknown n (%)	26 (36.6%)	39 (21.0%)
	Urological pathology n (%)	5 (7.0%)	5 (2.7%)
	Others n (%)	5 (7.0%)	21 (11.4%)
**Dialysis** **Method**	Conservative n (%)	10 (14.1%)	2 (1.1%)
	Hemodialysis n(%)	50 (70.4%)	168 (90.8%)
	Peritoneal n(%)	11 (15.5%)	15 (8.1%)
**Duration of dialysis (months)**		14 [9–21]	31 [18–48]
**Prior transplant**		1 (1.4%)	3 (1.6%)
**Panel Reactive Antibody (%)**		0 [0–0]	0 [0–0]
**Mismatches (n)**		3 [3–6]	3 [2–3]
**Donor age (year)**		42 ± 10	41 ± 13
**Donor–male gender n (%)**		39 (54.9%)	111 (60.0%)
**Deceased Donor Characteristics**			
**Cold ischemia time (hours)**		-	21.8 [19.3–23.4]
**Cause of donor death**	Cranial trauma (%)	-	80 (43.2%)
	Cerebrovascular (%) Other n (%)	- -	79 (42.7%) 26 (14.1%)
**Hypertension donor n (%)**		-	63 (34.1%)
**Donor creatinine (mg/dl)**		-	1.34 ± 0.76
**Kidney Donor Profile Index (KDPI)**		-	63 ± 24.1
**Induction**	Without n (%)	32 (45.1%)	0 (0%)
	Basiliximab n (%)	11 (15.5%)	148 (80.0%)
	Timoglobuline n (%)	28 (39.4%)	37 (20.0%)
**Immunosuppression**	Tacro+AZA+PDN	-	14 (7.6%)
	Tacro+MMF+PDN	62 (87.3%)	162 (87.6%)
	MMF+PDN	9 (12.7%)	-
	Tacro+SRL+PDN		9 (4.9%)
**Acute rejection**		19 (26.8%)	23 (12.6%)
**Cytomegalovirus**		13 (18.6%)	69 (38.1%)
**Kidney volume (cm**^**3**^**)**		153.7 ± 38.0	169.9 ± 52.9
**Adjusted kidney volume s/c (cm**^**3**^**/1.73m**^**2**^**)**		151.7 ± 41.7	167.7 ± 55.1
**Creatinine at discharge (mg/dl)**		1.37 ± 0.42	2.71 ± 1.18
**eGFR at discharge (ml/min)**		59.4 ± 19.0	28.3 ± 14.8
**One-year renal function**			
**One-year creatinine (mg/dl)**		1.24 ± 0.33	1.43 ± 0.56
**One-year eGFR (ml/min)**		64.85 ± 23.89	53.97 ± 26.50

Abreviations: AZA: azathioprine; Tacro: Tacrolimus; PDN: prednisone; MMF: mycophenolate; SRL: Sirolimus.

**Fig 1:**
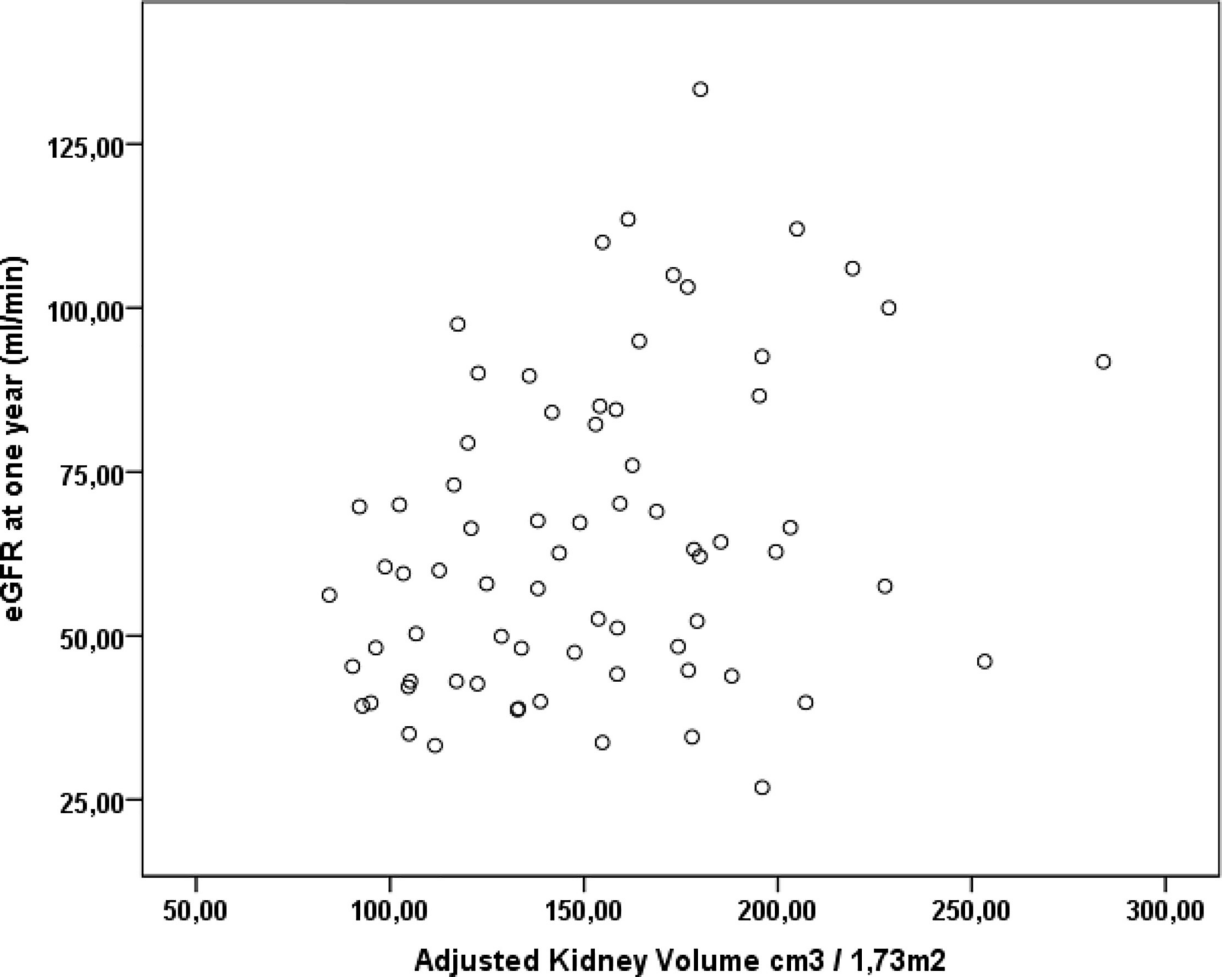
Correlation between adjusted kidney volume and glomerular filtration rate (eGFR) at 1 year in living donors (r = 0.311, p = 0.008).

Correlation analysis revealed a positive correlation between adjusted kidney volume and eGFR one year after transplantation (r = 0.311 and p = 0.008; “Fig 1”). The multivariate analysis indicated that the incidence of rejection and increased adjusted kidney volume were independent factors that predicted better renal function one year after transplantation (Table 2).

**Table 2:** Linear regression univariate and multivariate analysis–factors associated with improved kidney function after one year–living donor group.

Coefficients^a^					
Univariate	Unstandardized Coefficients		Standardized Coefficients	t	Sig.
	B	Std. Error	Beta		
Receptor age	-.553	.281	-.264	-1.972	.055
Receptor BMI (kg/m^2^)	1.106	.724	.218	1.528	.133
Adjusted kidney volume	.170	.082	.269	2.071	.044*
Panel	-.003	.146	-.003	-.022	.982
Dialysis method	.220	.235	.127	.934	.355
Donor age	-.173	.338	-.066	-.511	.612
Donor gender	-9.533	6.390	-.191	-1.492	.143
Baseline disease	-2.704	2.651	-.141	-1.020	.313
Induction therapy	.918	3.636	.034	.252	.802
Immunosuppression	-8.973	9.929	-.118	-.904	.371
Acute Rejection	-19.446	7.777	-.336	-2.500	.016*
Cytomegalovirus	-1.274	7.863	-.021	-.162	.872
**Multivariate**					
Rejection	-16.849	6.591	-.297	-2.556	.013*
Adjusted kidney volume	.176	.072	.285	2.452	.017*

Abbreviations: BMI—body mass index

The equation for predicting eGFR at the one-year follow-up in living donor is as follows:

eGFR1year=41.67−(16.85×Rejection)+(0.176×AdjustedKidneyVolume)

**Fig 2:**
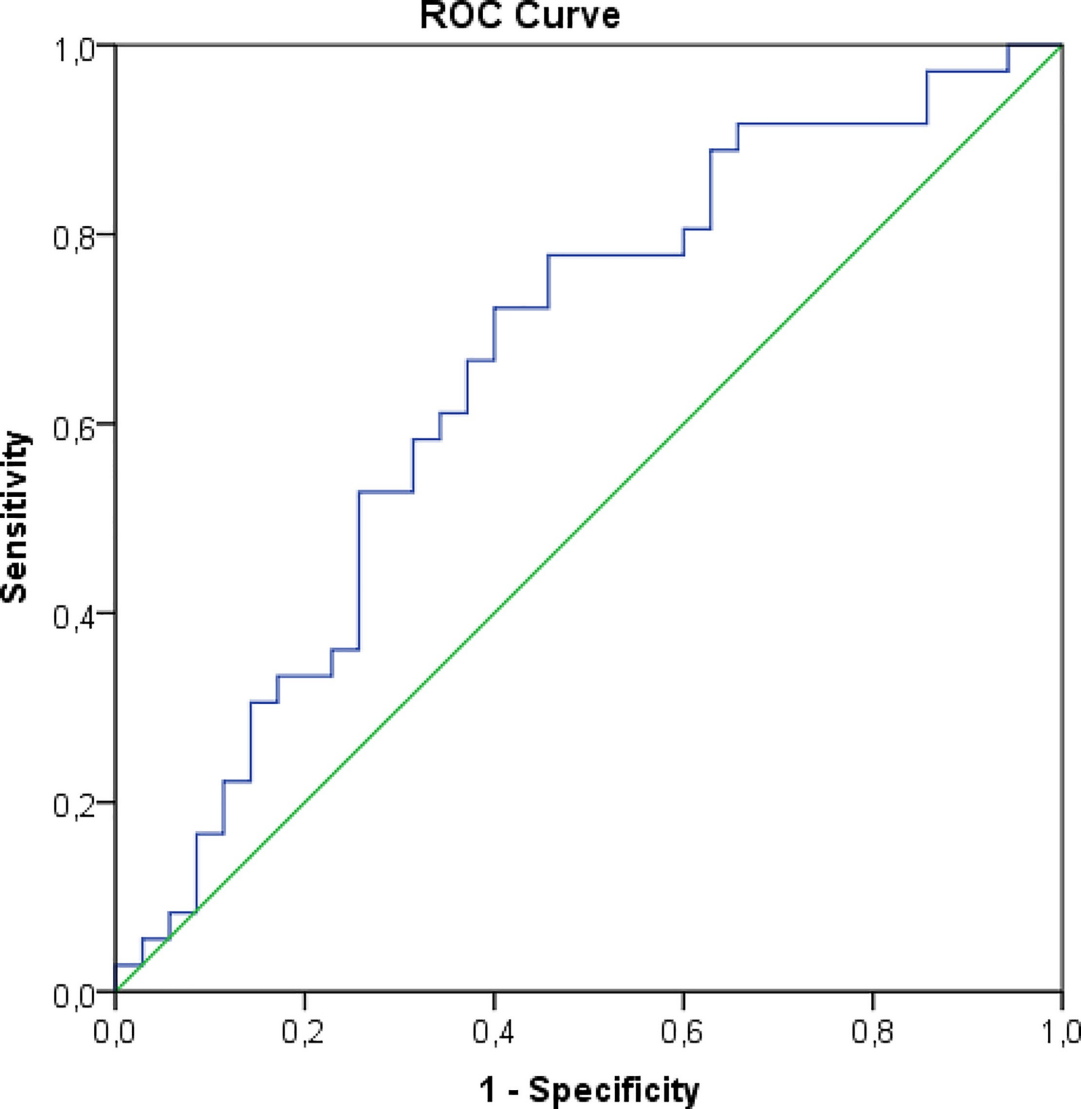
ROC curve between adjusted kidney volume and an estimate glomerular filtration rate (eGFR) at 1 year in living donors transplantation (curve area = 0.662, p = 0.019).

A ROC curve was generated using an estimated GFR higher than 60 ml/min as the reference and the adjusted kidney volume as the test variable. The area under the curve was 0.662 (p = 0.019) and the optimal cut-off value was 140.2 cm^3^ (72.2% sensitivity and 60.0% specificity; “Fig 2”). This value was correlated with a renal function of 71.8 ± 26.1 at the one-year follow-up (p = 0.005).

### Deceased donor transplantations

Among the 185 deceased donor transplantations, the mean recipient age was 50 ± 13 years; the recipients were predominantly white males. The most prevalent baseline diseases were arterial hypertension and diabetes. The patients had low immunological risk, with a median panel reactivity of zero. Induction therapy was performed using basiliximab in most cases. The predominant immunosuppressive regimen was the combination of tacrolimus, mycophenolate and prednisone. The rejection rate one year after transplantation was 12.6%, and the cytomegalovirus infection rate was 38.1%. The mean kidney volume adjusted for body surface area was 167.7 ± 55.1 cm^3^, with a mean GFR at the one-year follow-up of 53.97 ± 26.50 ml/min (Table 1).

**Fig 3:**
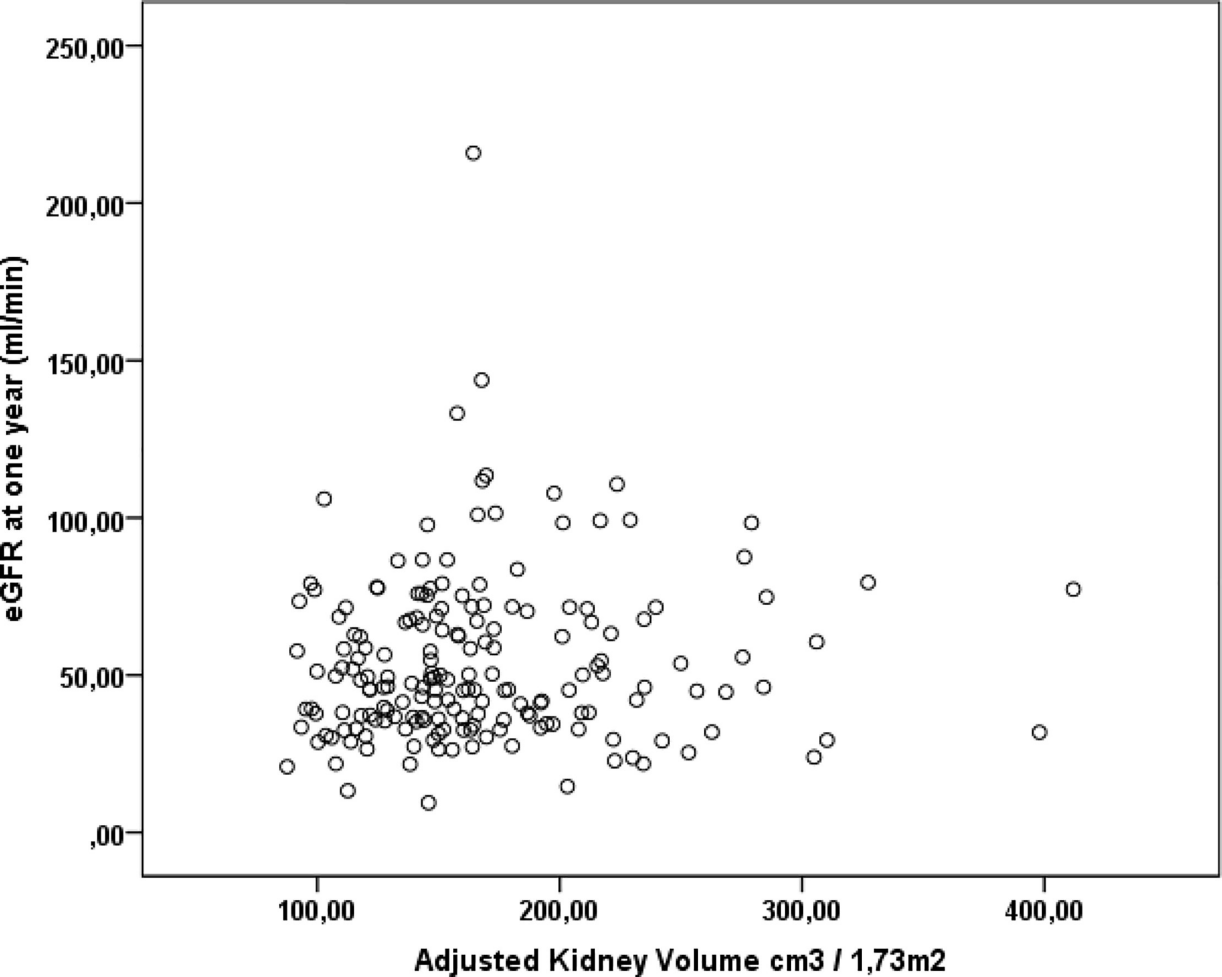
Correlation between adjusted kidney volume and estimated glomerular filtration rate (eGFR) at one year in deceased donors transplantations (r = 0.08, p = 0.279).

Correlation analysis revealed a positive correlation between adjusted kidney volume and eGFR one year after transplantation, with a non-significant difference (r = 0.08, p = 0.279; “Fig 3”). The multivariate analysis indicated that low KDPI, absence of rejection and high Adjusted Kidney volume were independent predictive factors for better renal function one year after transplantation. (Table 3).

**Table 3:** Linear regression—univariate and multivariate analysis of factors associated with better eGFR at the end of the first year in the deceased donor group.

	**Est.**	**S.E.**	**t val.**	**p**
(Intercept)	58.23	17.85	3.26	0.00
Receptor Age (years)	-0.05	0.16	-0.31	0.76
Receptor BMI (Kg/m2)	0.27	0.37	0.73	0.46
Adjusted Kidney Volume	0.09	0.03	2.57	0.01
Panel	0.16	0.11	1.40	0.16
Dialysis Type Peritoneal	3.13	6.61	0.47	0.64
Dialysis Type Preemptive	-14.63	17.72	-0.83	0.41
Disease Glomerulonephrithis	-10.10	6.47	-1.56	0.12
Disease Hypertension	0.68	4.87	0.14	0.89
Disease Others	-0.96	6.26	-0.15	0.88
Disease Undetermined	-4.64	5.39	-0.86	0.39
Disease Urological	10.17	11.31	0.90	0.37
Induction Tymoglobulin	-2.99	6.33	-0.47	0.64
Imuno:Tacrolimo+Aza+PDN	-7.53	7.25	-1.04	0.30
Imuno: Tacrolimo+SRL+PDN	-3.53	8.82	-0.40	0.69
Rejection	-13.20	5.34	-2.47	0.01
Cytomegalovirus	-1.04	3.80	-0.27	0.79
Cold Ischemia Time	0.35	0.50	0.70	0.48
Mismatch	-1.61	1.78	-0.91	0.36
KDPI	-0.38	0.08	-4.86	0.00
**Multivariate**	**Est.**	**S.E.**	**t val.**	**p**
(Intercept)	65.26	6.54	9.98	0.00
Adjusted Kidney Volume	0.08	0.03	2.30	0.02
Rejection	-12.93	5.09	-2.54	0.01
KDPI	-0.38	0.07	-5.36	0.00
BMI: body mass index; KDPI: Kidney Donor Profile Index				

The equation for predicting the eGFR at the one-year follow-up in deceased donor is as follows:

eGFR1year=65.2−(0.38×KDPI)−(12.93×Rejection)+(0.08×AdjustedKidneyVolume)

**Fig 4:**
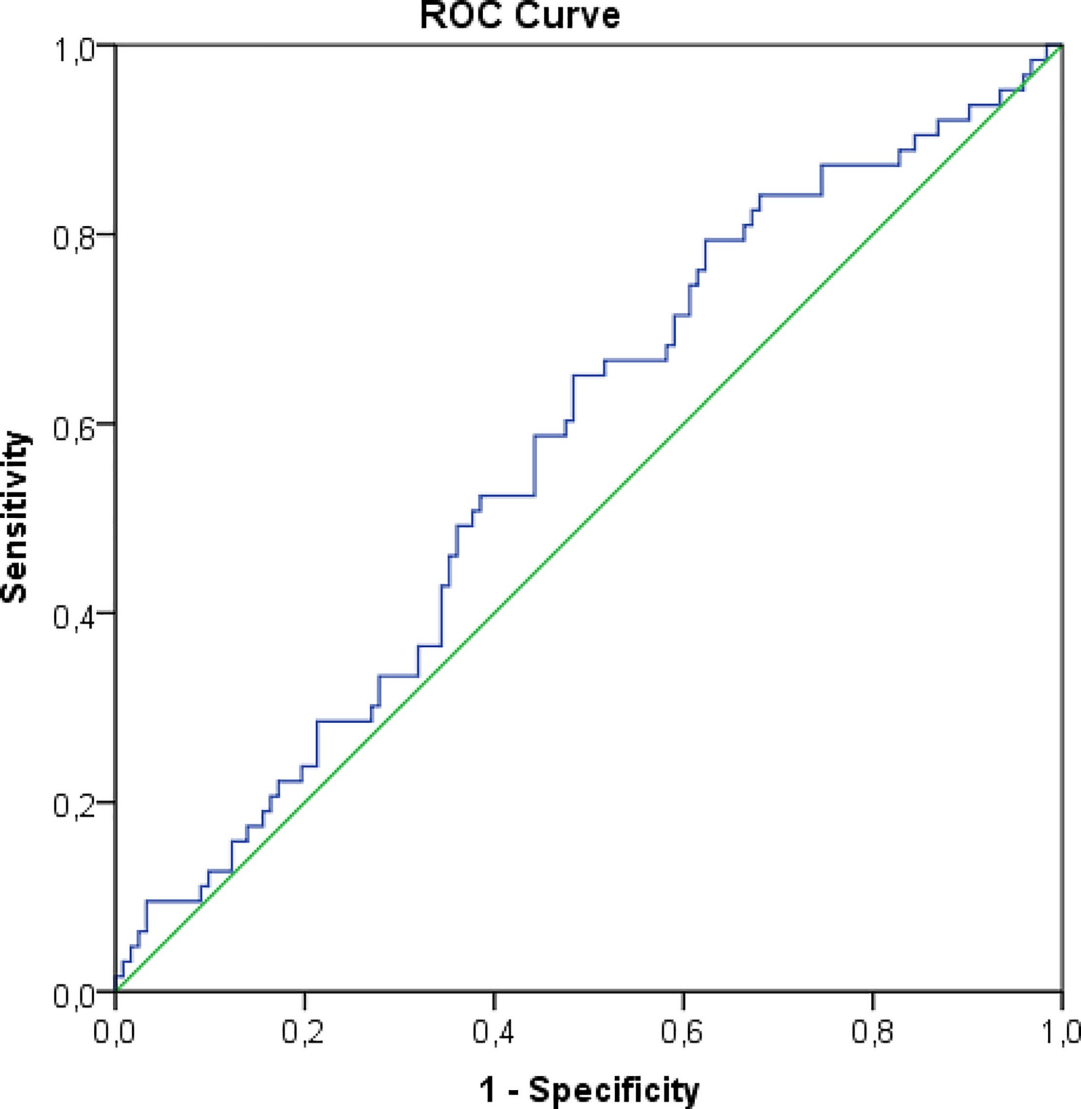
ROC curve between adjusted kidney volume and glomerular filtration rate (eGFR) after 1 year in deceased donors (curve area = 0.573, p = 0.1).

An ROC curve was generated using the same parameters as for the living donors. The area under the curve was 0.573 (p = 0.1); therefore, this result was not statistically significant (“Fig 4”). The optimal cut-off value was 140.9 cm^3^, with 79.4% sensitivity and 37.7% specificity. This value was correlated with a renal function of 56.9 ± 28.9 at the one-year follow-up (p = 0.023).

## Discussion

Kidney volume, which was estimated by measuring the dimensions of the kidney during the back-table surgery, was correlated with one-year renal function in living and deceased donor transplants. Furthermore, studies have suggested that grafts from men with a larger BMI compared with those of female recipients have superior survival rates [28,29].

One point to highlight is that graft volume estimates have already exhibited a good correlation with late renal function in living donor transplantations [10–17]. In a study by Poggio et al. [10], kidney volume measurements using helical 3D computerized tomography (CT) were correlated with renal function two years after living donor kidney transplantations. Similarly, Yano et al. [11] also found a positive correlation with three-year renal function when measuring kidney volume. In these previous reports, however, the authors did not evaluate other confounding factors associated with renal function in kidney transplantation. Furthermore, no other clinical indicators that are predictive of long-term renal function—including immunological risk, immunosuppression and acute rejection—have been described in association with kidney volume in the literature [10–17]. In our study, we showed that kidney volume is independently correlated with one-year renal function when considering several possible confounders. We developed a renal function prediction equation containing rejection occurrence and kidney volume as key factors. Additionally, a kidney volume less than 140 cm^3^ was associated with poorer one-year renal function, corroborating data from Nicholson et al. [18]. These authors demonstrated a positive correlation between kidney mass and improved five-year renal function when estimating kidney volume using ultrasound imaging. Contrarily, other authors [19,20] have failed to correlate kidney volume with renal function in transplantation, particularly in deceased donors.

In deceased donors, other variables, including donor characteristics and cold ischemia time, may also affect the analysis. This situation complicates the estimation of eGFR. Therefore, in this study we opted to separate the analysis on the basis of donor type. We found a direct correlation between kidney volume and eGFR in living donor transplantations. In deceased donors the effect of adjusted kidney volume in one-year kidney function was less pronounced (0.08 per kidney volume in deceased donors compared to 0.18 per kidney volume in living donors) but remains significantly. The donor´s characteristics summarized in KDPI also appears as an important predictable variable.

The validity of reproducible kidney size measurements for volume estimation in the present study is noteworthy. Previous studies have reported highly variable kidney size measurements, particularly those provided by organ procurement organizations (OPOs). In this case, the correlation between kidney size measurement provided by OPOs and kidney weight was weak (r = 0.41). As shown by our findings, kidney volume estimations using height, width and thickness measurements showed a strong correlation with kidney weight (r = 0.64).

The simple and rapid method for estimating kidney volume, which was determined using three simple measurements made by the surgeon during the back-table surgery, stands out as a strength of this study. Another important aspect that differentiated this study from others was the consideration of several possible confounders associated with one-year renal function.

There are several weaknesses associated with this study. First, it is a single-center study; there was intrinsic variability related to the method for evaluating kidney measurements. In addition, kidney volume should have been estimated using weight; however, we chose not to compare the data with organ weight due to the lack of a standardized procedure for removing the perigraft fat. Furthermore, we do not use calipers to measure the size of the kidney, and we did not use nuclear medicine or exogenous clearance to estimate renal function. This situation was related to the costs and time associated with those procedures, which render them not-easily-implementable in daily clinical practice. For this reason, the most commonly used tool to measure GFR is still the renal clearance estimated by equations. Despite these limitations, we used the MDRD 4, which shows a good correlation with iohexol plasma clearance (r = 0.769) [30].

In addition, we found a higher rate of acute rejection in living donor transplants. This was due to the lower use of induction therapy at the time of the study. However, the majority of the episodes of rejection were T-cell mediated (Banff IA) and promptly treated [26]. Besides, the adjustments made in the multivariate analysis may correct this effect and minimizes it over the outcome.

In conclusion, the kidney volume calculated using the ellipsoid formula was positively correlated with a better eGFR at one year in living and deceased donor transplantations. An adjusted kidney volume, lower than 140 cm3 was associated with worsened renal function.

## Supporting information

S1 Fig Measures used to estimate renal volume using a graduated ruler. A: Craniocaudal (length); B: laterolateral (width) and C: anteroposterior (thickness) measurements, expressed in centimetres (cm). The final graft volume was calculated using the three measurements taken and the ellipsoid formula.
(TIF)
