# Supplementary figures and images for: Impact of adjusted kidney volume measured in the bench surgery on one-year renal function in kidney transplantation
Source: PLoS One. 2019 Nov 4;14(11):e0224364. doi: 10.1371/journal.pone.0224364 (PMC6827889; doi:10.1371/journal.pone.0224364)

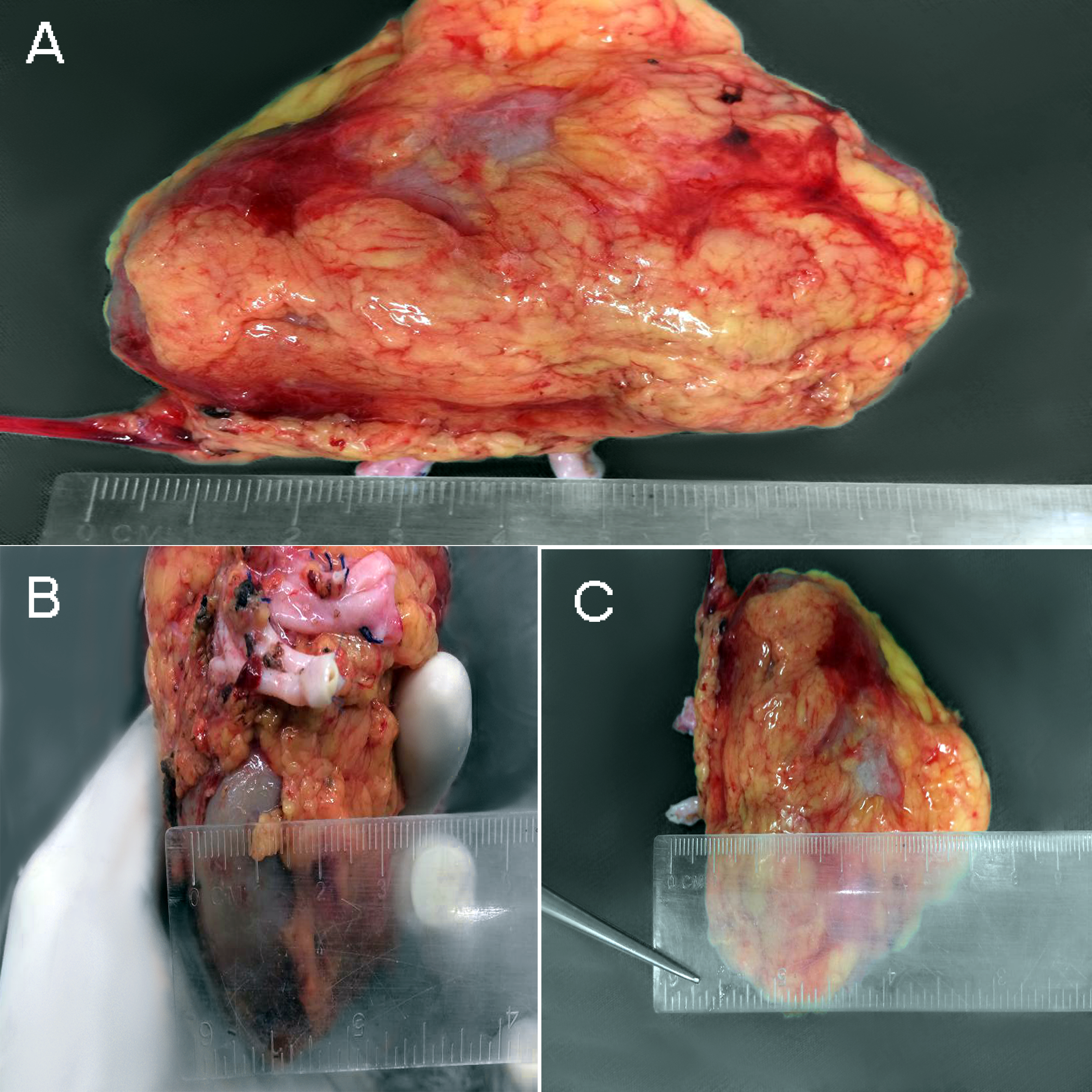

Supplement: S1 Fig — A: Craniocaudal (length); B: laterolateral (width) and C: anteroposterior (thickness) measurements, expressed in centimetres (cm). The final graft volume was calculated using the three measurements taken and the ellipsoid formula. (TIF) [file pone.0224364.s001.tif]
